# The protective role of selenium against dental amalgam-induced intracellular oxidative toxicity through the TRPV1 channel in DBTRG glioblastoma cells

**DOI:** 10.1590/1678-7757-2020-0414

**Published:** 2021-02-10

**Authors:** Derya CEYHAN, Kadriye Gorkem Ulu GUZEL, Bilal CIG

**Affiliations:** 1 Suleyman Demirel University Faculty of Dentistry Department of Pediatric Dentistry Isparta Turkey Suleyman Demirel University, Faculty of Dentistry, Department of Pediatric Dentistry, Isparta, Turkey.; 2 Adnan Menderes University Faculty of Dentistry Department of Pediatric Dentistry Aydın Turkey Adnan Menderes University, Faculty of Dentistry, Department of Pediatric Dentistry, Aydın, Turkey.; 3 Ahi Evran University Faculty of Medicine Department of Physiology Kirsehir Turkey Ahi Evran University, Faculty of Medicine, Department of Physiology, Kirsehir, Turkey.

**Keywords:** Apoptosis, Dental amalgam, Glioblastoma, Oxidative stress

## Abstract

**Objective:**

The exposure to mercury (Hg) from dental amalgams is a suspected causative factor in neurological diseases. This study investigated the toxic effects of two different amalgam compositions related to Hg and the protective effects of selenium against the toxic effects of Hg through the TRPV1 channel in the human DBTRG glioblastoma cell line.

**Methodology:**

Six groups of the cells were organized. Analyses of cell viability, apoptosis, caspase 3 and caspase 9 activities, mitochondrial membrane depolarization, reactive oxygen species (ROS) production, and Western Blotting for protein expression levels were performed.

**Results:**

Cell viability values were lower in amalgam with high copper (HCu) and low copper (LCu) groups independently of time but were increased by selenium and capsazepine (p<0.001 and p<0.05). Conversely, apoptosis rates, caspase 3 and caspase 9 expression, ROS formation, mitochondrial membrane depolarization, and protein expression levels were higher in the HCu and LCu groups but were decreased by selenium (p<0.001 and p<0.05).

**Conclusions:**

Selenium combined with an amalgam of either HCu or LCu decreases the toxic effects created by Hg in human DBTRG glioblastoma cells.

## Introduction

Dental amalgam contains approximately 50% elemental mercury (Hg^0^), the exposure to which from dental amalgams is a suspected causative factor in neurodevelopmental and neurodegenerative diseases.^1^ Accordingly, developing new approaches to clinical applications is essential to prevent exposure to the possible negative effects of Hg in dental amalgams.

The toxic effects of dental amalgam, which has been in use in dentistry for more than 150 years, and the possible side effects of Hg from dental amalgams have been the subject of several studies. Amalgam fillings were reported as safe for human health by many community and government organizations, and by scientific health associations between 1997 and 2009;^2^ however, in 2008, the US Food and Drug Administration stated that there may be neurotoxic effects associated with dental amalgams containing Hg that are dangerous to children and fetuses.^3^ At the United Nations Environment Program (UNEP) International Minamata Convention in 2013, provisions for the phasing down of the use of dental amalgam, the regulation of its usage, and the elimination of Hg or the effects of Hg from dental amalgams were developed.^4^ The 2015 report of the European Commission’s Scientific Committee on Emerging and Newly Identified Health Risks (SCENIHR) emphasized that, although current studies do not suggest preventing the use of amalgam fillings, the patients’ age, pregnancy, allergy and renal function status should be considered when making decisions regarding their application. SCENIHR also stated that further toxicity studies and the development of new alternative materials with high biocompatibility were necessary.^5^

Various forms of Hg exhibit a strong affinity to the thiol groups in cellular proteins or membranes and, upon linking to the thiol groups, block certain physiological and metabolic functions, damage the calcium (Ca^2^) homeostasis and redox equilibrium, and cause oxidative stress and apoptosis.^1,6^ The extrinsic cell-surface death receptor-dependent pathway and the intrinsic mitochondria-dependent pathway are well known apoptotic pathways.^7^ Additionally, oxidative stress caused by the endoplasmic reticulum induces apoptosis, essentially by an increase in intracellular Ca^2^ concentrations.^8^ Several ion channels of the plasma and intracellular membranes control cytosolic free Ca^2^ion concentrations. One non-selective cation channel of the transient receptor potential (TRP) channel superfamily of particular note is the transient receptor potential cation channel subfamily vanilloid member 1 (TRPV1), which responds to such stimuli as oxidative stress and capsaicin (CAP).^9,10^ The activation of TRPV1 showed a strong influx of external Ca^2^, Ca^2^ release from intracellular stores most likely included in the endoplasmic reticulum and an increase in the mitochondrial Ca^2^, and mediated Ca^2^-dependent cell death in mouse dorsal root ganglion neurons.^11^ Overloaded Ca^2^ entry induces the production of excessive intracellular reactive oxygen species (ROS) by an increase in mitochondrial membrane depolarization and apoptosis via the activation of caspase pathways.^12^

The essential trace element selenium (Se) plays a cofactor role in antioxidant enzyme glutathione peroxidase, and is involved in antioxidant defense by selenoproteins, protecting the cell membrane against oxidative damage. Se interacts with heavy metals, and Hg has a selenophilic property and shows a higher affinity for Se than for thiol groups.^1^ Previous studies investigating Se effect have reported a reduction in the cytotoxicity of amalgam, whereas others suggest otherwise.^13,14^ Selenoprotein P – a Se transport protein – is an important extracellular antioxidant that is essential for neuronal survival and function.^15^ Recent studies found that Se plays a modulator role on the TRPV1 channel in neuronal cells.^16,17^ Likewise, Se may reduce amalgam-induced toxicity through the modulation of TRPV1 channel activity.

In our study, we develop an alternative approach to the inhibition of Hg-related amalgam toxicity. To this end, the toxic effects of two different amalgam compositions were investigated, along with the Se protective effects against the Hg toxic effects through the TRPV1 channel in the human DBTRG glioblastoma cell line. The presence of the TRPV1 channel in the DBTRG glioblastoma cell line was reported as a finding of a recent study.^18^

## Methodology

### Cells and chemicals

This study was conducted on human DBTRG glioblastoma cells conceded by Dr. Laszlo Pecze, Department of Anatomy, Faculty of Medicine, Fribourg University, Fribourg, Switzerland. Chemicals were obtained from these sources: Dihydrorhodamine-123 (DHR123) and tris-glycine gel were from Molecular Probes (Eugene, OR, USA); 3-(4,5-dimethylthiazol-2-yl)-2,5-diphenyltetrazolium bromide (MTT) and caspase 3 substrate (AC-DEVD-AMC) were from Sigma-Aldrich (Madrid, Spain), and caspase 9 substrate (AC-LEHD-AMC) was from Bachem (Bubendorf, Switzerland); 5’,6,6’-tetrachloro-1,1’,3,3’-tetraethyl-benzimidazolylcarbocyanine iodide (JC-1) was from Santa Cruz Biotechnology (Dallas, TX, USA); caspase 3 and caspase 9 primary antibodies were from Cell Signaling Technology (Istanbul, Turkey)**;** secondary antibodies were from GE Healthcare (Amersham, UK); capsazepine (CpZ) and capsaicin (CAP) were from Santa Cruz Incorporated Company (Istanbul, Turkey); RPMI-1640 cell culture medium, 4-(2-Hydroxyethyl)piperazine-1-ethanesulfonic acid, N-(2-Hydroxyethyl)piperazine-N′-(2-ethanesulfonic acid) (HEPES), 3-[(3-chomalidopropyl)dimethylammonio]-1-propanesulfonate (CHAPS), dithiothreitol (DTT), Ethylenediaminetetraacetic acid (EDTA), dimethyl sulfoxide (DMSO), ethylene glycol-bis[2-aminoethyl-ether]-*N,N,N,N*-tetraacetic acid (EGTA), and sodium selenite were from Sigma-Aldrich Chemical (St. Louis, MO, USA). Before an analysis, the reagents were equilibrated for 30 min at room temperature. Non-gamma-2 amalgams with low (11.9%) and high (24%) Cu alloy components were purchased from Ankara, Turkey (Cavex Holland BV, Haarlem, The Netherlands). Non-gamma-2 amalgams increase the corrosion resistance of traditional amalgams and are developed by increasing the copper (Cu) content in the dust.

### Preparation of amalgams

The pre-dosed amalgam capsules were mixed using a high-energy mixer (Silamat S5, Ivoclar Vivadent AG, Liechtenstein) for 5–7 seconds, in accordance with the manufacturers’ instructions, and then transported to the cell culture media according to the groups.^19^ The weight of a pre-dosed amalgam capsule was 0.4 g.

### Cell culture

Human DBTRG glioblastoma cells were passaged using a Roswell Park Memorial Institute (RPMI) 1640 medium, and Penicillin Streptomycin (1% Pens.St.) and fetal bovine serum (10% FBS) were added to the cell culture medium. The cells were then cultured in a liquid jacketed incubator (37ºC and 5% CO_2_), designed for cell cultures to investigate the dental amalgam toxicity. The cells were grown until 90% confluent in flasks (250 ml, 75 cm^2^) and were counted and equalized before analysis in all groups (Casy Modell TT, Roche, Germany). Considering the increased food consumption due to the proliferating cells, the media were changed twice a week and Pens.St. was increased to 2% to reduce the risk of contamination.

### Groups

The cells in all groups were counted using an automatic cell counter to achieve 1×10^6^ cells per flask, which were divided into six main groups (Table 1).^17,20^

**Table 1:** Study groups according to the cell culture mediums

Group	Cell culture medium
Control	Same cell culture medium for periods of 2, 12, 24 and 48 h without any incubation with CAP, CpZ and Se
Se	Sodium selenite (200 nM) for 24 h^17^
Amalgam with low Cu (LCu)	Amalgam with low Cu for periods of 2, 12, 24 and 48 h^20^
Amalgam with high Cu (HCu)	Amalgam with high Cu for periods of 2, 12, 24 and 48 h^20^
Amalgam with low Cu + Se (Se+LCu)	Sodium selenite (200 nM) for 24 h and then treated with amalgam with low Cu for periods of 2, 12, 24 and 48 h
Amalgam with high Cu + Se (Se+HCu)	Sodium selenite (200 nM) for 24 h and then treated with amalgam with high Cu for periods of 2, 12, 24 and 48 h

For the analyses, the cells were further treated with CAP (0.01 mM, channel-specific agonist) to activate the TRPV1 channel, and, when necessary, were inhibited with the TRPV1 channel blocker CpZ (0.1 mM, channel-specific antagonist).^21^ CAP and CpZ were dissolved in DMSO for the preparation of the stock solution, with phosphate-buffered saline (PBS) used for dilution, and the pH was adjusted. The amalgams were added directly to the cell culture medium. The stock solution was prepared in sterile distilled water for Se (0.2 mM) and diluted (10^6^times) to achieve the final concentration. After incubation, the control and treated cells were examined for cell viability, caspase 3 and caspase 9, apoptosis, intracellular ROS production and mitochondrial membrane depolarization, and for Western Blotting analyses.

### Cell viability assay (MTT)

For the assessment of Se protective effects on cell viability, the mitochondrial activity of living cells was evaluated by the quantitative colorimetric assay of MTT. The cells were plated in 96-well culture plates, and following the amalgam and Se treatments, the cells were washed and incubated with MTT (0.5 mg/ml) stain for 90 min at 37ºC. In the following stage, the supernatant was discarded and DMSO was added to dissolve the formazan crystals. The absorbance of each well was measured for 490 and 650 nm wavelengths by a microplate reader (Infinite Pro200; Tecan Austria GmbH, Groedig, Austria). The experiments were performed in triplicate for cell viability assay (n=3/group), and the data were presented as a fold increase and compared with the control group.

### Caspase 3, caspase 9 and apoptosis assays

A previously reported method was used to identify caspase 3 and caspase 9 activity.^22,23^The stimulated or resting cells were sonicated, and cell lysates were incubated with 2 ml of substrate solution (20 mM HEPES, pH 7.4, 2 mM EDTA, 0.1% CHAPS, 5 mM DTT, and 8.25 mM of caspase substrate) for 2 h at 37°C. Caspase 3 substrate (AC-DEVD-AMC) and caspase 9 substrate (AC-LEHD-AMC) cleavages were read using a multi-well (96-well culture plates) reader device (Infinite Pro200; Tecan Austria GmbH, Groedig, Austria) at 360 nm excitation and 460 nm emission wavelengths. The obtained caspase expression values were the quantitative fluorescence readings recorded by the instrument, and these amounts were estimated as fluorescence units/mg protein. Data were presented as a fold increase and compared with the control group.

The apoptosis assay was quantified using a commercial kit (Cell-APOPercentage Apoptosis Assay, Biocolor Ltd., Northern Ireland) in accordance with the manufacturer’s instructions (https://www.biocolor.co.uk/product/cell-apopercentage-apoptosis-assay/). The assay uses a dye that is selectively imported by cells undergoing apoptosis, and, since necrotic cells cannot retain the dye, they are not stained. The detection of apoptosis by spectrophotometer is possible by actively transporting the APOPercentage dye into the cells and staining the apoptotic cells red when the membrane of an apoptotic cell loses its asymmetry. The absorbance of apoptosis dye was measured at 550 nm in the microplate reader (Infinite Pro200; Tecan Austria GmbH, Grodig, Austria). The experiments were performed in triplicate for the caspase 3, caspase 9 and apoptosis assays (n=3/group).

### Intracellular ROS measurement

ROS were measured using DHR123 dye in the microplate reader (Infinite Pro200; Tecan Austria GmbH, Grodig, Austria), as reported in a previous study.^24^ Non-fluorescent DHR123 dye is indicative of ROS when oxidized to cationic Rh123 localizing in mitochondria and giving green fluorescence. The cells (10^6^cells/ml) were washed with a serum-free RPMI-1640 medium, incubated with 0.02 mM DHR123 at 37ºC for 25 min, and then washed in PBS. The fluorescence intensity of Rh 123 was measured in the microplate reader. The excitation for Rh123 was read at wavelengths of 488 nm and the emission was 543 nm. The experiments were performed in triplicate for intracellular ROS analysis (n=3/group). The values were expressed as a fold increase and compared with the control group.

### Mitochondrial membrane potential (JC-1) determination

JC-1, a cationic dye, is an assay method for the measurement of mitochondrial membrane depolarization. The cells were incubated at 37ºC for 15 min with the mitochondrial membrane potential marker JC-1 (1μM), as previous.^25^ For the green JC-1 signal, the excitation and emission values were measured at 485 nm and 535 nm wavelengths, respectively; whereas for the red JC-1 signal, the excitation and emission values were measured at 540 nm and 590 nm wavelengths, respectively. A microplate reader (Infinite Pro200) was used to analyze fluorescence changes. The experiments were performed in triplicate for the JC-1 determination assay (n=3/group), and the values were presented as a fold increase and compared with the control group.

### Western Blotting

Standard Western Blotting procedures were used.^26^ The frozen cells were homogenized in the lysis buffer to determine intergroup variations of β-actin (polyclonal antibody), poly (ADP-ribose) polymerase 1 (PARP1) (polyclonal antibody), and active caspase (split caspase) 3 (p17-specific Polyclonal Antibody) and 9 (p35/p10 Polyclonal Antibody) protein expression levels. The cells were then centrifuged at 16000 g for 20 min and the supernatant was collected. A Bradford (595 nm wavelength) reagent was used for the assessment of total protein, and the resulting bands were visualized with ECL Western HRP Substrate (Millipore Luminate Forte, USA) and X-ray film (GE Healthcare, Amersham Hyperfilm ECL, UK) and normalized against the β-actin protein. The Western Blotting experiments were performed in triplicate (n=3/group), and the data were presented as a fold increase and compared with the control group.

### Statistical analysis

The analysis of the data was conducted using the Statistical Package for Social Sciences (SPSS, version 17.0; SPSS, Chicago, Illinois, USA) software. The study results were expressed as mean±standard deviation (SD), and a Mann-Whitney U test and analysis of variance (ANOVA) were conducted. A post-hoc Tukey’s multiple comparison test was run following the ANOVA. The accepted statistical significance limit was p<0.05.

## Results

### Cell viability (MTT) values

**Figure 1:**
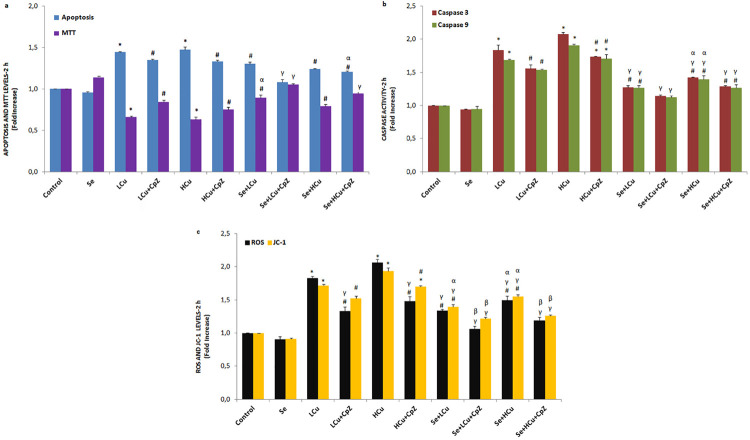
Effects of Se, LCu and HCu (2h) on apoptosis and MTT (a), caspase 3 and caspase 9 (b), and intracellular ROS and JC-1 (c) levels (mean±SD and n=3/group). Values are presented as a fold increase (experimental/control). Statistical differences were assessed by ANOVA with Tukey’s post-hoc testing (*p˂0.001 and #p˂0.05 versus control and Se groups. γp˂0.001 and #p˂0.05 versus LCu and HCu groups. αp˂0.05 and γp˂0.001 versus LCu+CpZ and HCu+CpZ groups. βp˂0.05 versus Se+LCu and Se+HCu groups)

**Figure 2:**
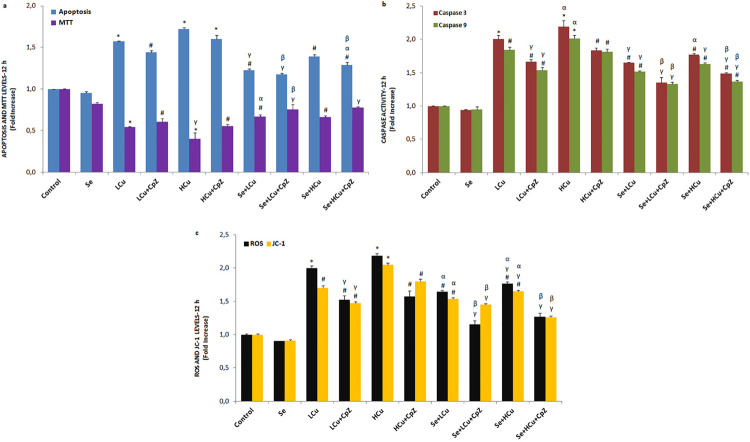
Se, LCu and HCu incubations (12h) for apoptosis and MTT (a), caspase 3 and caspase 9 (b), and intracellular ROS and JC-1 (c) levels (mean±SD and n=3/group). Values are presented as a fold increase (experimental/control). Statistical differences were assessed by ANOVA with Tukey’s post-hoc testing (*p˂0.001 and #p˂0.05 versus control and Se groups. γp˂0.001 and #p˂0.05 versus LCu and HCu groups. αp˂0.05 and γp˂0.001 versus LCu+CpZ and HCu+CpZ groups. βp˂0.05 versus Se+LCu and Se+HCu groups)

**Figure 3:**
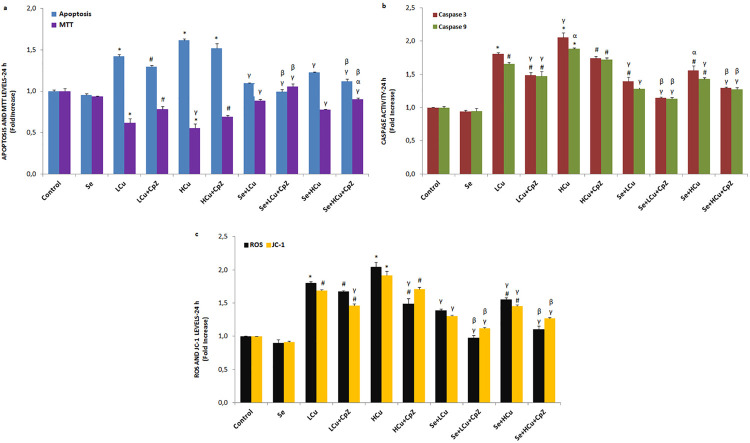
Effects of Se, LCu and HCu (24h) on apoptosis and MTT (a), caspase 3 and caspase 9 (b), and intracellular ROS and JC-1 (c) levels (mean±SD and n=3/group). Values are presented as a fold increase (experimental/control). Statistical differences were assessed by ANOVA with Tukey’s post-hoc testing (*p˂0.001 and #p˂0.05 versus control and Se groups. γp˂0.001 and #p˂0.05 versus LCu and HCu groups. αp˂0.05 and γp˂0.001 versus LCu+CpZ and HCu+CpZ groups. βp˂0.05 versus Se+LCu and Se+HCu groups)

**Figure 4:**
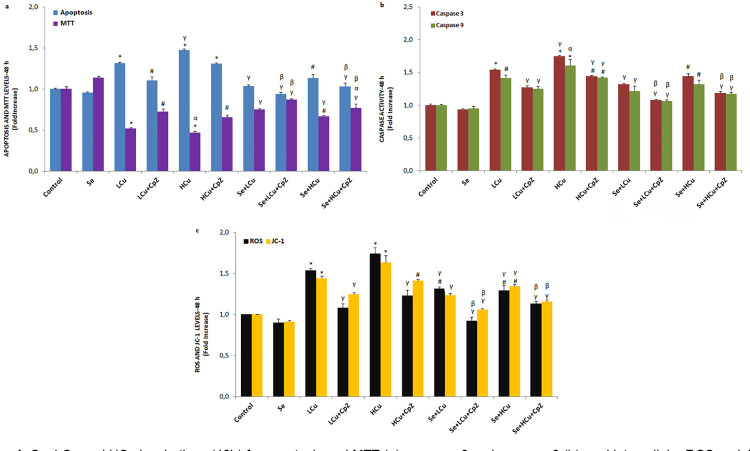
Se, LCu and HCu incubations (48h) for apoptosis and MTT (a), caspase 3 and caspase 9 (b), and intracellular ROS and JC-1 (c) levels (mean±SD and n=3/group). Values are presented as a fold increase (experimental/control). Statistical differences were assessed by ANOVA with Tukey’s post-hoc testing (*p˂0.001 and #p˂0.05 versus control and Se groups. γp˂0.001 and #p˂0.05 versus LCu and HCu groups. αp˂0.05 and γp˂0.001 versus LCu+CpZ and HCu+CpZ groups. βp˂0.05 versus Se+LCu and Se+HCu groups)

Figures 1, 2, 3 and 4 show the MTT values in the control, Se, LCu, HCu, Se+LCu and Se+HCu groups at 2, 12, 24 and 48 h, respectively. The MTT levels of the LCu and HCu groups were significantly lower than those of the control and Se groups (p<0.001 and p<0.05), although their levels were increased in the Se+LCu and Se+HCu groups by the Se treatments (p<0.001 and p<0.05). Cell viability levels were further increased in the Se+LCu+CpZ and Se+HCu+CpZ groups by CpZ treatments (p<0.001 and p<0.05). There were no changes in the MTT levels among the 2, 12, 24 and 48 h groups.

### Caspase 3 and caspase 9 expression values and apoptosis rates

The caspase 3 and caspase 9 expression levels and apoptosis rates in the six groups at 2, 12, 24 and 48 h are shown in Figures 1, 2, 3 and 4, respectively. The caspase 3 and caspase 9 expression values and apoptosis rates of the LCu and HCu groups were significantly higher than those of the control and Se groups (p<0.001 and p<0.05). In comparison with the LCu and HCu groups, the Se+LCu and Se+HCu groups exhibited significant decreases in caspase 3 and caspase 9 expression levels, as well as in apoptosis rates (p<0.001 and p<0.05). CpZ treatments further decreased caspase 3 and caspase 9 expression levels and the apoptosis rates in the Se+LCu and Se+HCu groups (p<0.001 and p<0.05). The caspase 3 and caspase 9 expression levels were lower in the 48 h groups when compared to the 2, 12 and 24 h groups.

### Intracellular ROS and JC-1 values

The intracellular ROS and JC-1 values in the six groups at 2, 12, 24 and 48 h are presented in Figures 1, 2, 3 and 4, respectively. The intracellular ROS levels of the LCu and HCu groups were significantly higher than in the control and Se groups (p<0.001 and p<0.05). In the six groups, a decrease over time was noted in the intracellular ROS and JC-1 values. Se and CpZ pre-treatments contributed to the decrease in the intracellular ROS and JC-1 levels in the LCu and HCu groups (p<0.001 and p<0.05).

### Procaspase 3 and procaspase 9 and PARP1 expression values

**Figure 5:**
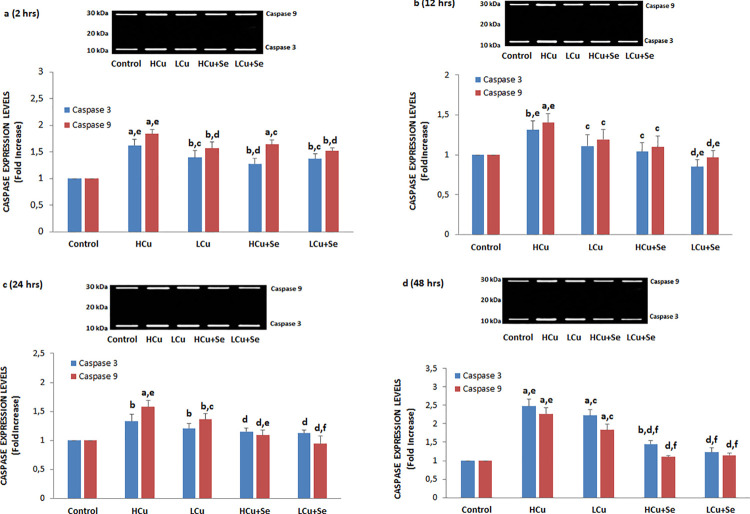
Effects of Se, LCu and HCu [2 (a), 12 (b), 24 (c) and 48 (d) h] on cleavage caspase 3 and caspase 9 expression levels (mean±SD and n=3/group). Values are presented as a fold increase (experimental/control). Statistical differences were assessed by ANOVA with Tukey’s post-hoc testing (ap˂0.001 and bp˂0.05 versus control. cp˂0.05 and dp˂0.001 versus HCu group. ep˂0.05 and fp˂0.001 versus LCu group)

In apoptotic processes, the conversion to cleavage (active) caspase 3 and caspase 9 leads to a decrease of procaspase 3 and procaspase 9 expression levels.^27^ Active caspase 3 and caspase 9 expression levels becoming a determinant for apoptotic pathways were analyzed in human DBTRG glioblastoma cells at 2, 12, 24 and 48 h (Figure 5) in our study. The active caspase 3 and caspase 9 expression levels in the LCu and HCu groups were significantly higher than in the control group (p<0.001 and p<0.05). In comparison with the LCu and HCu groups, the caspase 3 and caspase 9 expression levels were decreased in the Se+LCu and Se+HCu groups (p<0.001 and p<0.05).

**Figure 6:**
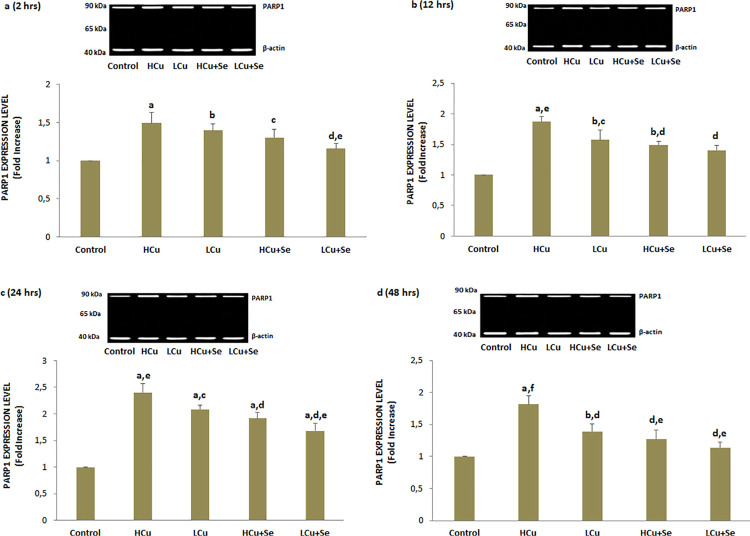
Se, LCu and HCu incubations [2 (a), 12 (b), 24 (c) and 48 (d) h] for PARP1 expression level (mean±SD and n=3/group). Values are presented as a fold increase (experimental/control). Statistical differences were assessed by ANOVA with Tukey’s post-hoc testing (ap˂0.001 and bp˂0.05 versus control. cp˂0.05 and dp˂0.001 versus HCu group. ep˂0.05 and fp˂0.001 versus LCu group)

The PARP1 enzyme is activated in response to oxidative DNA breaks, and targets the damaged DNA zones.^28^ In the six groups, the damage related to the DNA repair mechanisms was determined from PARP1 activities (Figure 6). The PARP1 expression level of the LCu and HCu groups was significantly higher than in the control group (p<0.001 and p<0.05). The PARP1 expression level was lower in the Se+LCu and Se+HCu groups when compared with the LCu and HCu groups (p<0.001 and p<0.05).

## Discussion

**Figure 7:**
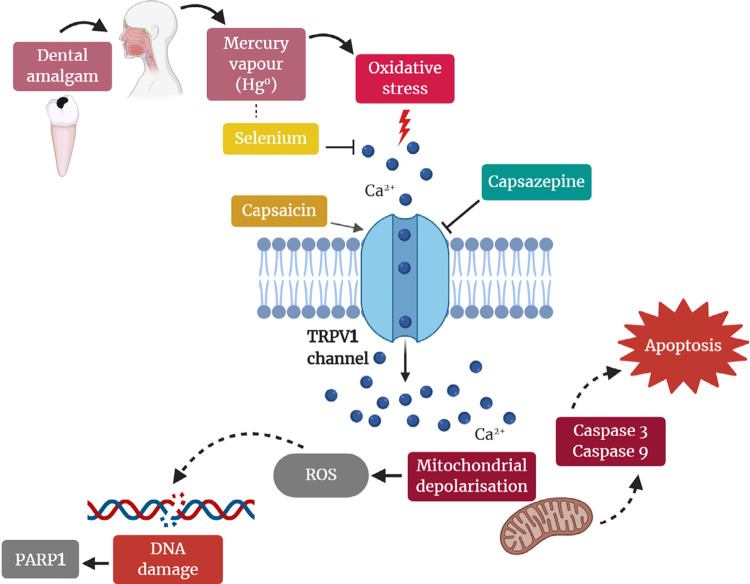
Possible molecular pathways of involvement of selenium (Se) on oxidative stress, Ca2+ influx and apoptosis through the TRPV1 channel in dental amalgam-Hg-induced DBTRG glioblastoma cell line. Reactions of mercury (Hg0) from dental amalgam with cellular molecules result in oxidative stress. The TRPV1 ion channel of intracellular membranes is activated by oxidative stress and capsaicin (CAP) and blocked by capsazepine (CpZ). Increased intracellular Ca2+ through the TRPV1 channel induces the reactive oxygen species (ROS), mitochondrial membrane depolarization, and apoptosis via activation of caspase pathways. Se forms Se-Hg complexes, decreases Ca2+ influx, mitochondrial oxidative stress and apoptosis through the modulation of the cell TRPV1 channel activity and inhibits Hg-induced toxicity. Created with BioRender.com

In our study, we investigated the Hg-related toxic effects of two different amalgam compositions and Se protective effects against the Hg toxic effects through the TRPV1 channel in the human DBTRG glioblastoma cell line. We found that Se acted as an anti-apoptotic agent, decreasing ROS formation and Ca^2^entry through the TRPV1 channel (Figure 7). To the best of our knowledge, there has been no previous study reporting the Se effects on cell viability, caspase 3 and caspase 9 activities, apoptosis rates, intracellular ROS and JC-1 values, and procaspase 3 and procaspase 9 and poly (ADP-ribose) polymerase 1 (PARP1) expression levels through the TRPV1 channel in human DBTRG glioblastoma cells over the evaluation of toxicity of dental amalgams containing Hg.

Hg is classified as a harmful toxic heavy metal that has no known physiological role in the human body, which then lacks effective mechanisms to excrete it.^15^Hg elemental (Hg^0^), inorganic (Hg^1^), and organic (methyl, ethyl, phenyl compounds of Hg) forms exist in the environment, and these forms can evolve into each other in both the environment and the human body.^1^ Hg^0^ vapor from dental amalgam is absorbed by the respiratory tract, and is distributed around the body in the bloodstream. Bioaccumulation occurs especially in the liver, kidneys and brain.^29^ The uncharged monoatomic form of Hg^0^ vapor provides it with a highly diffusible and lipid soluble characteristic that can easily breach the blood-brain barrier and the lipid bilayers of cells and cell organelles such as mitochondria, before the cells oxidize it into an inorganic form (Hg^2^).^15,30^ Hg^2^reacts with such intracellular molecules as enzymes, glutathione, ion channels and transporters. The activities of such molecules are inhibited and normal cellular functions are affected by these reactions, and oxidative stress increases.^15^The TRPV1 ion channel of intracellular membranes is activated by oxidative stress, and results in increased cytosolic free Ca^2^ and apoptotic cell injury.^16,17,21^ Moreover, metallic Hg vapor and methyl Hg compounds permeate through the central nervous system and induce toxic effects easier and more frequently than inorganic Hg compounds.^31^ We tested two different amalgam compositions in the human DBTRG glioblastoma cell culture medium and observed the toxic effect created by Hg^0^, and found the effect to be decreased by a Se antioxidant element.

Treatment with antioxidants, including Se and Ca^2^ channel blockers, can protect against Hg cytotoxicity, as well as methylmercury and mercury chloride neurotoxicity.^6,13^ For example, researchers have found that Se reduces the cytotoxic effects of inorganic and organic Hg compounds and dental amalgam.^13,32^ Se forms Se-Hg complexes and inhibits Hg-induced toxicity. Several mechanisms, such as Hg sequestration, anti-oxidative activity, thiol glutathione synthesis, elevated glutathione peroxidase activity, increased selenoprotein concentrations and detoxification by demethylation show how Se compounds eliminate Hg-induced toxicity.^1^ Methylmercury has been reported to elevate intracellular Ca^2^ concentrations through by the promotion of extracellular Ca^2^ entry and Ca^2^release from intracellular stores.^33^ Methylmercury and mercury chloride have been shown to increase Ca^2^and ROS formation in cerebellar granule cell cultures, and mercury chloride induces apoptosis in a Ca^2^ entry-dependent way.^6,34^ Ca^2^ increases may also be triggered by ROS production, and ROS formation is involved in the Ca^2^ homeostasis disruption.^6^ Mitochondria are the main intracellular targets for ROS generation, and it has been shown that mitochondria are damaged at very low doses of Hg in rats.^35^ Hg compounds attach to thiol groups, and cause a depletion of sulfhydryl proteins and glutathione that damage the mitochondria, resulting in the formation of free radicals and oxidative stress.^15^Ca^2^ channel blockers inhibit ROS formation and Ca^2^ growth,^6^ which means that blocking the TRPV1 channel that responds to CAP and oxidative stress by Se and CpZ facilitates the inhibition of exaggerated Ca^2^influx into cells exposed to dental amalgam, and thus prevents cell damage. In our study, ROS production and mitochondrial depolarization levels increased in human DBTRG glioblastoma cells in the amalgam with LCu or HCu groups. The decreases in ROS production and mitochondrial membrane depolarization levels over time were further enhanced by Se and/or CpZ treatments. We show here that reducing TRPV1 channel activity using Se and CpZ treatments had a significant effect on both Ca^2^homeostasis and redox equilibrium, and dental amalgam-induced toxicity was eliminated by the decrease of mitochondrial oxidative stress and apoptosis (Figure 7).

MTT assay, which is an assessment method of the cytotoxicity of biomaterials, was used to test the effects of dental amalgam on the survival of human DBTRG glioblastoma cells. Our results found that Hg from the amalgam in the LCu or HCu groups induced cell death in human DBTRG glioblastoma cells, regardless of time, although the cell viability in these groups was increased by Se and/or CpZ. Cell death induced by toxins can occur by apoptosis.^20^ The lack of effect of time on MTT values could be attributed to the fact that the Hg release remained at certain levels after causing acute damage to the cells according to the test times. That is, the cells suffered acute damage, but the damage did not progress. This finding concurs with the previously reported finding that freshly prepared amalgam is more cytotoxic than aged amalgam.^14^Accordingly, aged amalgams should be examined to determine the toxic effects of chronic exposure in terms of clinical relevance.

Organic and inorganic Hg species affect mitochondrial functioning by initiating alterations in the permeability of mitochondrial membranes and the release of a proapoptotic molecule – cytochrome c. Thus, in human T-lymphocytes, Hg species can induce an apoptotic cascade.^36^ There have been previous studies reporting the apoptotic effects of organic and inorganic Hg species on different cell types.^20,37,38^ Apoptosis is a cell death mode that leads to significant morphological and molecular challenges within the surrounding tissues, and various biochemical and physiological pathways, such as a sequence of cytosolic cysteine protease activation, occur at apoptosis. The activation of cytosolic cysteine proteases causes caspase activation, resulting in apoptosis.^7^ In the our study, although caspase 3 and caspase 9 activities and apoptosis were observed in human DBTRG glioblastoma cells in the amalgam with LCu or HCu groups, caspase 3 and caspase 9 expression and apoptosis levels were decreased by Se and/or CpZ treatments. This result refers to the higher Se affinity of Hg, the cofactor role of Se and the antioxidant defense mechanisms by selenoproteins, and the inhibition of ROS formation and Ca^2^ increase (Figure 7). We evaluated different periods of time to determine the time-dependent toxicity of Hg from dental amalgams. It was expressed that delayed Hg deposition in the lysosomes over time led to enhanced cytotoxicity.^38^On the other hand, the cytotoxicity of amalgam clearly decreased over time due to the combined effects of surface oxidation and further amalgamation. Oxidation on the amalgam surface leads to the dissolution of elements within the amalgam, while the further amalgamation fixes the residual Hg in the amalgam.^14^ Our noted decreases in apoptosis rates, caspase 3 and caspase 9 expression, intracellular ROS production and mitochondrial depolarization values after 12 hours suggest that the first 12 hours following the placement of dental amalgam into the mouth are important, since time-dependent toxicity emerged and a detoxification mechanism developed from metallothioneins over time. This finding can be attributed to the increased evaporation of Hg from the amalgam during setting, meaning that acute exposure to Hg has more toxic effects on one’s health than chronic exposure.

In tissues, Hg is found to be attached to such thiol-containing molecules as cysteine, glutathione and metallothionein. The most common intracellular metal-binding proteins are metallothioneins – from the cysteine-rich protein family. Metallothioneins have a high affinity for Cu, which plays an important role in the regulation of metallothionein synthesis. However, Hg has a potential for metallothionein induction and can interact with Cu.^1^ Although pure Cu has been reported to be cytotoxic and increased Cu concentrations in amalgams have been thought to enhance their cytotoxic effects, low-Cu and high-Cu amalgams have been shown to have the same cytotoxicity level.^14^ Cu ratio effect on amalgam toxicity was also considered in our study, with amalgams with different Cu ratios chosen for study; however, no difference between the groups was found, in consistence with previous studies. Secondary changes in DNA due to the attachment of Hg to thiol groups may be important. Mitochondrial dysfunction induced by Hg have been shown to increase lipid peroxidation and the oxidation of proteins and DNA, and epigenetic changes, such as DNA methylation, and DNA strand breakage have been linked to Hg compound exposure.^15,39^ DNA damage^40^ and the initiation of genotoxic processes^41^ using methylmercury have been demonstrated. When PARP1 expression levels were considered, amalgams with LCu or HCu caused DNA damage to human DBTRG glioblastoma cells, but Se decreased the extent of the damage.

Our study has some limitations. For example, we used 200nM sodium selenite; however, different Se concentrations may have different effects. Furthermore, the amount of Hg released from dental amalgams was not measured, although the determination of Hg release from amalgams over time is important to determine the effects of acute and chronic toxicity in more detail. Moreover, to the analyses performed, a qualitative evaluation with fluorescence microscopy would further support the findings. Further studies are required to address these issues.

Dental amalgam is still in use today due to its cost, mechanical properties and clinical indications, and the potential toxic effects of other materials. Se decreases the toxic effects associated with Hg, according to the results of our study. Moreover, Se deficiency may be observed in patients with amalgam restorations, since Hg forms a compound with Se in the body. In this respect, our study can guide future investigations into the creation of strategies involving Se application as a supplement to patients that undergo amalgam restorations.

## Conclusion

We found that Se has an anti-apoptotic effect on human DBTRG glioblastoma cells, decreasing Ca^2^ influx, mitochondrial oxidative stress and apoptosis by the modulation of the cell TRPV1 channel activity. Se may thus be useful as an anti-apoptotic agent, since it decreases Hg toxic effects in human DBTRG glioblastoma cells when combined with an amalgam of either HCu or LCu. The production of amalgams with Se content can prevent toxicity by limiting Hg release, although further laboratory studies are needed to produce such materials.
